# Research Progress and Future Development Trends in Medicinal Plant Transcriptomics

**DOI:** 10.3389/fpls.2021.691838

**Published:** 2021-07-28

**Authors:** Junda Guo, Zhen Huang, Jialing Sun, Xiuming Cui, Yuan Liu

**Affiliations:** ^1^Faculty of Life Science and Technology, Kunming University of Science and Technology, Kunming, China; ^2^Yuxi Walvax Biotechnology Co., Ltd., Yuxi, China; ^3^Yunnan Provincial Key Laboratory of Panax Notoginseng, Kunming, China; ^4^Key Laboratory of Panax Notoginseng Resources Sustainable Development and Utilization of State Administration of Traditional Chinese Medicine, Kunming, China; ^5^Kunming Key Laboratory of Sustainable Development and Utilization of Famous-Region Drug, Kunming, China

**Keywords:** medicinal plant, transcriptomics, RNA-Seq sequencing, functional genes, secondary metabolism, developmental mechanism

## Abstract

Transcriptomics is one of the most popular topics in biology in recent times. Transcriptome sequencing (RNA-Seq) is a high-throughput, high-sensitivity, and high-resolution technique that can be used to study model and non-model organisms. Transcriptome sequencing is also an important method for studying the genomes of medicinal plants, a topic on which limited information is available. The study of medicinal plants through transcriptomics can help researchers analyze functional genes and regulatory mechanisms of medicinal plants and improve breeding selection and cultivation techniques. This article analyzes and compares the applications of transcriptome sequencing in medicinal plants over the past decade and briefly introduces the methods of transcriptome sequencing and analysis, their applications in medicinal plant research, and potential development trends. We will focus on the research and application progress of transcriptome sequencing in the following four areas: the mining of functional genes in medicinal plants, development of molecular markers, biosynthetic pathways of secondary metabolites, and developmental mechanisms of medicinal plants. Our review will provide ideas for the mining of functional genes of medicinal plants and breeding new varieties.

## Introduction

Currently, there are more than 10,000 medicinal plant species known in China. However, several medicinal plants are non-model plants, owing to the lack of genomic information and insufficient research on their functional genes and genetic mechanisms (Liu et al., 2019). Therefore, it is essential to apply transcriptome sequencing to medicinal plants. With the advent of the post-genomic age, proteomics, genomics, and transcriptomics are now recognized as popular approaches. Among these, transcriptomics is currently the most extensively researched and applied discipline, as it enables the investigation of functional as well as differentially expressed genes (DEGs) (Jia et al., 2015). In medicinal plant research, transcriptomics and genomics have several key differences. First, in plant research, genome assembly is more complex and expensive than transcriptomic analysis; the transcriptome can be used to monitor the overall transcriptional activity of an organism without a reference genome. Second, the transcriptome is altered depending on the time and space of observation, as it not only reflects the differences in gene expression at different temporal and spatial points, but also harbors information on secondary metabolic pathways. Studies have shown that owing to the different growth environments and growth periods of medicinal plants, even among the same species, gene expression patterns reflect time- and space-based differences, resulting in the unique accumulation patterns of medicinal components. Therefore, the transcriptome is more suitable for identifying genes related to medicinal components in medicinal plants (Wang et al., 2009).

Different plant tissues and organs exhibit temporal and spatial differences in gene expression. These differences are of significant importance to research on functional gene mining in medicinal plants, construction of gene regulatory networks, secondary metabolites, resistance genes, and genetic diversity. In recent years, with the steady advancements in sequencing technology, transcriptome research tools have evolved from the traditional chip hybridization platform to RNA sequencing technology (RNA-Seq) (Mironova et al., 2015). This method can be used to perform whole transcriptome analysis without a genomic reference sequence and offers advantages such as high throughput, digital signals, and high sensitivity. Transcriptome sequencing technology is a widely used sequencing method in the field of molecular biology (Sun and Wei, 2018). To date, this technology has been used extensively in model plants such as maize (Xu B. et al., 2014), Arabidopsis (Guo et al., 2009), and rice (Li et al., 2012). This article discusses transcriptome sequencing technology and sequencing analysis approaches and summarizes the progress in the application of transcriptome sequencing in four aspects of medicinal plant research: functional gene mining, molecular marker development, identification of secondary metabolite biosynthetic pathways, and identification of developmental mechanisms in medicinal plants, to provide ideas for the functional gene mining of medicinal plants and breeding of new varieties.

## Application of Sequencing Technology

### Next-Generation Sequencing Technology

The traditional Sanger sequencing method is time-consuming, laborious, and expensive. However, the development of next-generation sequencing (NGS) in 2006 fulfilled several key requirements of researchers in the field of molecular biology. NGS lacks the shortcomings of the first-generation sequencing technology and is a low-cost, rapid, high-throughput, and deep-coverage technique. Compared to traditional sequencing technology, NGS can simultaneously sequence millions of nucleic acid molecules, thereby facilitating the analysis of the transcriptome and genome of any species. Currently, NGS is used to assemble multiple model and non-model plant and animal genomes to obtain all gene sequences of a certain species, such as SARS-CoV-2 (Li W. W. et al., 2020) and *Chosenia arbutifolia* (Chen et al., 2014; Mei et al., 2016; Feng et al., 2019). NGS is primarily used for the sequencing and analysis of mRNA and small RNA in the transcriptome. Some common NGS platforms include the Roche 454, Illumina Solexa, and ABI SOLiD (Zhang et al., 2016) (Supplementary Data Sheet 1).

Roche 454 sequencing technology was released in 2005 and is a high-throughput sequencing platform based on pyrosequencing (Margulies et al., 2005). Compared to other technologies, 454 sequencing technologies have the advantages such as a long-read length and rapid running speed. This method does not require fluorescent labels or nucleic acid probes; however, it may introduce deletion or insertion errors during the sequencing process (Liang et al., 2017). Solexa sequencing technology is based on the principle of sequencing by synthesis, through the random attachment of DNA fragments to the flow cell. Following extension and amplification, several million clusters are formed on the glass surface, with each cluster containing several thousand identical DNA molecular fragments. Fluorescently labeled dNTPs are sequenced when the DNA strand is extended. This platform can be used in genome-wide expression studies without the need for reference sequences or synthetic probes (Liu et al., 2012). The primary disadvantages of this platform are the short read length and the difficulty in assembling the reads from scratch. Solid sequencing technology was released in 2007 and is a massively parallel sequencing technology based on the use of magnetic beads. This technology can be used for the large-scale amplification and high-throughput parallel sequencing of single-copy DNA fragments based on the continuous ligation and synthesis of fluorescently labeled oligonucleotides (Li and Xu, 2019). The high accuracy is the greatest advantage of this technology, whereas the greatest disadvantage is that once an error occurs, it is prone to chain decoding errors.

### Third-Generation Sequencing Technology

Advances in sequencing technologies have enabled the development of the third-generation sequencing. As NGS technologies require PCR amplification among other processes, template migration, base mismatches, and GC preferences are common challenges associated with these methods, which adversely affect the accuracy and completeness of sequencing results. Third-generation sequencing has been widely used in genome sequencing, structural variation detection, transcriptome sequencing, and methylation detection owing to the long-read length. PacBio single-molecule real-time (SMRT) sequencing technology and Oxford Nanopore sequencing technology (Ma et al., 2019) are two third-generation sequencing methods (Supplementary Data Sheet 1).

The SMRT sequencing technology is based on the principle of sequencing by synthesis. Compared with traditional methods, SMRT technology has two novel characteristics. First, the fluorescent group is attached to the phosphoric acid group to address the issue of background noise. Second, amplification is not necessary, and SMRT enables more accurate quantification by self-correction (Li Y. M. et al., 2018). However, SMRT sequencing technology has several limitations, including the introduction of random errors. At present, the accuracy of a single read can be increased to 99.8% using the CCS sequencing method and by increasing the sequence coverage (Wenger et al., 2019). Nanopore single-molecule sequencing technology is a new-generation single-molecule real-time sequencing technology, which primarily uses changes in electrical signals to estimate the base composition. Nanopore sequencing has advantages such as high throughput, long-read length, and low cost. However, it also has drawbacks such as a high single-base error rate and random error.

Traditional methods for transcriptome data acquisition and analysis include expressed sequence tag (EST), complementary DNA-amplified fragment length polymorphism (cDNA-AFLP), hybridization technology-based chip technology (such as cDNA chip), serial analysis of the gene (also known as SAGE), and massively parallel signature sequencing (Cheng, 2004; Xu and Wu, 2005; Hang and Peng, 2006; Zhang and Sheng, 2008; Wu et al., 2009; Yang et al., 2009; Simkin et al., 2011; Qian et al., 2012; Zhang et al., 2012; Zhuo et al., 2015; Su et al., 2016; Supplementary Data Sheet 2). In the absence of a reference genome sequence, the above-mentioned methods can be expensive and time-consuming. Therefore, with the development of high-throughput sequencing technology, RNA-Seq technology based on the NGS and the third-generation sequencing technology is used as the mainstream method of transcriptome research.

### Transcriptome Assembly and Function Annotation Methods

**FIGURE 1:**
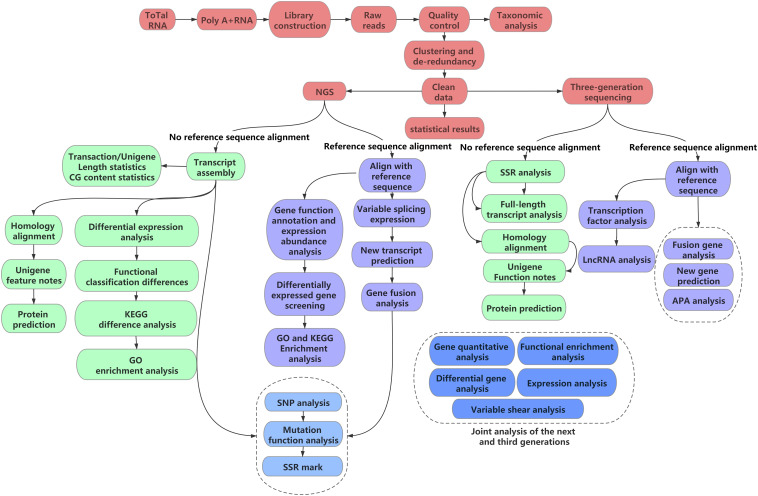
Flow chart of transcriptome analysis.

Transcriptome assembly is integral for various subsequent analyses. Owing to the considerably large quantity of transcriptome data, raw transcriptome data are error-prone (Nagalakshmi et al., 2008). Therefore, the selection of assembly software based on different transcriptomic data and research purposes is essential. The first point to consider is whether a reference sequence is available; based on this, assembly can be divided into two types: *de novo* assembly and reference sequence-based assembly (Figure 1). As most medicinal plants are non-model organisms, the corresponding genome sequence information is often lacking; therefore, reference assembly cannot be performed. *De novo* assembly is the only assembly method suitable for non-model plants.

Software packages for genome sequence-based assembly include Cufflinks (Trapnell et al., 2013) and Scripture (Guttman et al., 2010), and those for *de novo* assembly include Trinity (Grabherr et al., 2013), SOAPdenovo-*Trans* (Xie et al., 2014), Rnnotator (Martin et al., 2010), and Oases (Schulz et al., 2012). Among the four popular software packages used for *de novo* assembly, Trinity and SOAPdenovo-Trans use a single K-mer assembly method, whereas Rnnotator and Oases use a multiple K-mer assembly method. Although multiple K-mers can be used to obtain more transcript data, there is considerable redundancy generated when the results of multiple K-mers are merged, which increases the error rate and data complexity. Therefore, a single K-mer assembly method can be used more accurately (Strickler et al., 2012). SOAPdenovo-Trans uses an unknown base N to connect to the contig. This method reduces the quality of the assembly result, does not generate longer transcripts, and exhibits an average accuracy rate lower than that of the three other software packages (Lu, 2013). At present, Trinity is the primary *de novo* assembly software used, which can obtain higher-quality assembly results while ensuring accuracy and operating efficiency. Among genome sequence-based assembly software, Cufflinks adopts a conservative strategy assembly method, whereas Scripture adopts a sensitive strategy assembly method. The assembly quality of Cufflinks is better than that of Scripture, but the number of transcripts obtained with Scripture far exceeds that obtained with Cufflinks. With high-quality reference sequences, Cufflinks can obtain assembly results with greater accuracy and better quality.

Gene function annotation involves the use of bioinformatics methods for the comparison of unknown gene sequences with those available in public databases to determine the functions associated with each gene based on available data. At present, there are two methods primarily used for gene function classification: Gene Ontology (GO) function classification and Kyoto Encyclopedia of Genes and Genomes (KEGG) function classification. Among these, GO is the international standard method for gene function classification. It comprises a set of real-time updated controlled vocabulary to describe the genes of organisms and the properties of gene products in a holistic manner, including molecular functions, biological processes, and cellular components. The databases commonly used for annotating unigene sequences include the Non-Redundant Protein Sequence Database, GO, KEGG, Clusters of Orthologous Groups for Eukaryotic Complete Genomes, NCBI Nucleotide Sequence Database, Clusters of Orthologous Groups of Proteins, and Swiss-Prot database (Liu F. X. et al., 2018).

## Applications of Transcriptome Sequencing Technology in Medicinal Plant Research

### Mining Functional Genes of Medicinal Plants

Throughout their evolution, medicinal plants have developed various regulatory mechanisms to counter external stresses to adapt to their environments. Functional gene mining involves the identification of associated biosynthetic pathways, genes encoding key enzymes, and plant regulatory mechanisms, which helps improve our understanding of plant molecular biology. Xu W.R. et al. (2014) used the next-generation high-throughput sequencing technology (Illumina GA-II sequencing technology) to sequence the *Vitis amurensis* transcriptome and reported that 6,850 transcripts are involved in cold regulation. Among them, 3,676 upregulated and 3,147 downregulated transcripts and 38 major TF families involved in cold regulation were identified. This result provides the basis for further research on the cold tolerance mechanisms of *Vitis* species and the genes involved in the cold stress regulatory network. Pragati et al. (2018) used Illumina paired-end sequencing technology to sequence the transcriptome of the root and leaf tissues of *Aloe vera* and obtained 161,733 and 221,792 transcripts and 113,062 and 141,310 unigenes, respectively. Sixteen genes related to the biosynthesis of saponins, lignin, anthraquinone, and carotenoids were identified. This is the first *Aloe vera* transcriptome database and is of great significance for further research on genes related to the biosynthesis of important secondary metabolites in *Aloe vera* and other *Aloe* species and their metabolic regulation and the functions of specific genes in plant biology and physiology. Guo et al. (2018) performed transcriptome sequencing and analysis of *Paeonia suffruticosa* and obtained 81,725 unigenes, which may be related to the drought resistance mechanism. The role of plant hormone signaling pathway genes in the drought response was speculated, which formed the foundation for further research on the drought stress response mechanism of *P. suffruticosa*. Singh et al. (2017) used Illumina paired-end sequencing technology to sequence the *Trillium govanianum* transcriptome, obtained 69,174 transcripts, and identified a series of genes involved in the biosynthesis of steroid saponins and other secondary metabolite pathways. In a study on the biosynthesis of brassinosteroids, carotene, diterpenoids, flavonoids, phenylpropane, steroids, and terpenoids, leaf and fruit tissues were found to serve as the primary sites of steroidal saponin biosynthesis. This finding provided resources for genetic manipulation for the identification of potential biologically active metabolites and may help develop functionally related molecular marker resources. Loke et al. (2017) sequenced the transcriptome of *Polygonum minus* and obtained 188,735 transcripts. Furthermore, they reported 163,200 (86.5%) similarity matches of *P. minus* transcripts, most of which were with Arabidopsis (58.9%). Certain enriched metabolite pathways have been identified in the root and leaf tissues. These results will help further develop the genetic resources of this species. Li et al. (2016) used the Illumina sequencing method to sequence *Callerya speciosa*, obtained 161,926 unigenes, and identified 4,538 DEGs. DEGs related to a light signaling, starch synthesis, and cell wall loosening may be associated with the formation of storage roots. These findings provided data for subsequent research on the root development of *C. speciosa*, metabolites with medicinal value, and breeding. Hou et al. (2018) used NGS technology to study the transcriptome of leaf and fruit tissues of *Cornus officinalis* and identified 56,392 unigenes, with 4,585 significant DEGs. Among them, 1,392 genes were upregulated and 3,193 genes were downregulated in fruit tissues. Most DEGs are related to terpenoid biosynthesis and the regulation of secondary metabolism. This provides a basis for understanding plant gene expression and biosynthetic pathways. Rosmarinic acid is a multifunctional phenolic biologically active compound with antibacterial, antiviral, and antioxidant activities. Li et al. (2017) analyzed the transcriptome of *Dracocephalum tanguticum* and obtained 151,463 unigenes. In all, 22 rosmarinic acid-related biosynthetic genes were predicted, providing references for future research on biosynthetic genes related to rosmarinic acid in this species (Supplementary Data Sheet 3).

### Development of Molecular Markers Based on Transcriptome Sequencing

Simple sequence repeat (SSR) markers, also known as microsatellite DNA markers, are one of the most commonly used microsatellite markers. The core sequence of the tandem repeat is of one to six base pairs, and dinucleotide repeats are the most common sequences. The high polymorphism in these markers were attributed to the difference in the number of tandem repeats. Owing to the high polymorphism, simple operation, codominance, easy detection, and wide coverage, SSRs have been widely used in genetic map construction, genetic diversity analysis, gene mapping, and identification of parental relationships. Kapoor et al. (2020) used SSR markers to study the genetic diversity of *Asparagus* varieties from different areas of production in northwest India. A total of 122 alleles were amplified, ranging from three to eight, with an average of five alleles for each marker, and the size of the amplified alleles ranged from 90 to 680 bp. Genetic diversity analysis showed that most *Asparagus* varieties exhibit a conservative genetic background, and only accessions of *A. adscendens* are divided into two different groups, which indicates the broad genetic basis of this species compared to that of other species. These results are of considerable significance for hybrid breeding and preservation of *Asparagus* species in the future. Bhandari et al. (2020) used Illumina paired-end sequencing technology to develop a new microsatellite marker for *Salvadora oleoides*. They successfully designed 14,552 SSR markers from the 21,055 microsatellite repeats detected, and randomly selected and verified a subset of 7,101 SSRs; 94 primers were successfully amplified, 34 of which exhibited polymorphisms. This study provided a basis for further research on *S. oleoides*. Dinh et al. (2020) used the Illumina HiSeq 4000 sequencing platform to analyze the transcripts (from roots, leaves, and stems) of *Populus alba*. A total of 11,343 EST-SSRs were detected, and 101 primer pairs were screened from 7,774 pairs for polymorphism verification. Of these, 20 pairs of primers successfully amplified DNA fragments, and obvious polymorphisms were identified in the population. This finding was considered particularly important for the formulation of effective species conservation, restoration, and management strategies. Wang Y. S. et al. (2020) used NGS technology for transcriptome sequencing and analysis of *Gastrodia elata* and identified 34,322 unigenes. Among them, 2,007 (5.85%) unigenes contained at least one SSR. Among these SSRs, the AG/CT repeat motif was the most frequent, with a total of 498 (21.67%) detections. The findings of this study provided a deeper understanding of molecular mechanisms underlying the metabolism, growth, and development of *G. elata*. Lade et al. (2020) analyzed the genetic diversity and population structure of 96 *Tinospora cordifolia* samples collected from 10 different geographic regions in India and identified 7,611 SSRs from 268,149 transcripts. Tc131, Tc31, Tc129, Tc38, Tc16, Tc59, Tc60, Tc17, Tc106, and Tc130 were found to exhibit potential diversity. SSR-18, TCSSR-37, TCTSSR-59, TCTSSR-92, TCTSSR-123, and TCTSSR-126 served as potential markers. These materials and the newly developed SSR markers serve as valuable resources for further genetic improvement of *T. cordifolia*. Hina et al. (2020) used Illumina transcriptome sequencing technology and performed *de novo* assembly to obtain thorough genetic knowledge of two *Menispermum* species. A total of 53,712 and 78,921 unigenes were generated, and 521 polymorphic EST-SSRs were identified. The newly developed EST-SSR marker also exhibited high transferability among the tested *Menispermum* species. The new microsatellite markers will help investigate the population genetics of *Menispermum*. He et al. (2020) used microsatellite software to perform SSR site analysis of *P. lactiflora* and obtained 86,195 unigenes; 21,998 SSR sites were found distributed among 17,567 unigenes. Among the 100 pairs of randomly selected primers, 45 amplified clear polymorphic bands. These 45 primer pairs were used for cluster analysis of 16 *P. lactiflora* varieties. The new SSR molecular marker is helpful for genetic diversity research on *P. lactiflora* and molecular marker-assisted breeding (Supplementary Data Sheet 4).

### Exploring the Biosynthetic Pathways of Secondary Metabolites

Usually, secondary metabolites are the active components of medicinal plants, which not only have medicinal value, but also play an integral role in the adaptation of plants to the environment and resistance of plants to external stress. In different growth environments, growth stages, and organs, there are specific differences in the accumulation of secondary metabolites. Transcriptome sequencing is used to study the biosynthetic pathways of secondary metabolites and mine biosynthesis-related genes in different environments, growth stages, and organs. The data obtained can form the scientific basis for information on the accumulation and efficient utilization of active components in medicinal plants. *Entada phaseoloides* is an important traditional medicinal plant. Owing to its wind-dampness-eliminating effect and significant anti-inflammatory potential, the stems of *E. phaseoloides* are widely used in traditional medicine. Triterpene saponins are the primary biologically active compounds in *E. phaseoloides*. Liao et al. (2020) conducted comparative transcriptome analysis of the root, stem, and leaf tissues of *E. phaseoloides* and identified 26 cytochrome P450 and 17 uridine diphosphate glycosyltransferase candidate genes related to triterpene saponin biosynthesis. The findings contributed to investigations on the functional genomics of triterpene saponin biosynthesis. *Lantana camara* is an economically important essential oil-producing plant and a useful source of biologically active compounds, such as steroids, flavonoids, and phenylpropanoid glycosides. Shah et al. (2020) used transcriptome sequencing technology to perform *de novo* transcriptome analysis of the leaves and roots of *L. camara*. A total of 72,877 and 513,985 unigenes were obtained, with 229 and 943 genes involved in phenylpropanoic acid biosynthesis in leaf and root tissues, respectively. *Tetrastigma hemsleyanum* extract is used as a broad-spectrum antibiotic material for the treatment of fever and sore throat. Yan et al. (2020) conducted a comprehensive analysis of the metabolome and transcriptome of purple and green leaves of *T. hemsleyanum*. A total of 209 metabolites and 4,211 transcripts were differentially expressed in the purple and green leaves, and 16 compounds were found to be significantly associated with 14 transcripts involved in the anthocyanin biosynthesis pathway. *Saussurea lappa* has a significant pharmacological potential, primarily owing to the sesquiterpene lactones it produces. Bains et al. (2019) used NGS technology to sequence the leaf transcriptome of *S. lappa* and identified transcripts encoding proteins related to sesquiterpene and flavonoid biosynthesis. A relatively small number of transcripts encoded by genes related to the alkaloid pathway have been discovered. The findings contribute to studies on the functional genomics of this plant. To study genes related to isoflavone biosynthesis, Wang et al. (2018) conducted in-depth transcriptome sequencing of *Arisaema heterophyllum Blume* and obtained 35,686, 43,363, and 47,783 unigenes from the root, tuber, and leaf tissues, respectively. Eighty-seven candidate genes encoding isoflavone biosynthesis-related enzymes were identified, analyzed, and experimentally verified. This study provides a basis for further research on the pharmacological mechanisms of *Arisaema*. Flavonoids have a high medicinal value owing to their applicability in the treatment of various diseases, such as dengue, HIV, and cancer, as well as their antioxidation and anti-inflammatory properties. *Ginkgo biloba* leaves are enriched in flavonoids. Wu et al. (2018) performed transcriptome sequencing of *G. biloba* with different flavonoid contents and obtained 37,625 unigenes. Among the genes identified, several candidate genes are related to the biosynthesis, transportation, and regulation of flavonoids. The authors identified 14 genes enriched in flavonoid transport, one MYB gene encoding a transcription factor, and one dihydroflavonol-4-reductase gene involved in the flavonoid pathway. The findings led to the expansion of the existing *G. biloba* gene database, improved the scope of analysis of *Ginkgo* species, and provided valuable information for *Ginkgo*-related pharmaceuticals. Yuan et al. (2018) used NGS technology to perform *de novo* transcriptome sequencing and analysis of *Abrus mollis* leaves and studied the biosynthetic pathways of flavonoids and the related precursors. Liu M.M. et al. (2018) performed RNA sequencing of the leaf, root, and stem tissues of *Artemisia argyi* and identified 99,807 unigenes, including multiple genes encoding important enzymes or transcription factors related to the synthesis of terpenoids. The findings form the basis for functional research on the molecular mechanisms prevalent in *A. argyi*. Jayakodi et al. (2014) performed transcriptome sequencing of *Panax ginseng* root tissues using the 454 sequencing technology and found that cytochrome P450 and UDP-glycosyltransferase genes are involved in the biosynthesis of triterpene saponins. Liu et al. (2015) performed transcriptome analysis on the adventitious roots of *P. notoginseng*; 17% transcript differences were found in adventitious roots compared to that in common roots, and 21 genes related to ginsenoside synthesis were also identified. Jayakodi et al. (2015) conducted the transcriptome analysis on the leaves, roots, and flowers of *P. ginseng* and identified 107,340 unigenes, including 9,908 genes involved in 135 different metabolic pathways and 270 genes involved in the biosynthesis of triterpene saponins. Luo et al. (2011) analyzed the transcription levels of four *P. notoginseng* root samples and found that 32 genes were specifically expressed in the roots of annual ginseng, seven genes were specifically expressed in 6-year-old ginseng roots, and 38 genes were found to be involved in the biosynthesis of triterpene saponins (Supplementary Data Sheet 5).

### Transcriptomics and Developmental Mechanisms in Medicinal Plants

Transcriptomics has been used to study the differences in gene expression in medicinal plants under abiotic stress and to identify genes that affect the growth and development of medicinal plants and resistance to external stress. This information can help identify the key influencing factors in the growth and development process, provide a basis for the cultivation and breeding of medicinal plants, and facilitate the targeted selection of better varieties. Rastogi et al. (2019) conducted transcriptome sequencing of leaves under cold, drought, waterlogged conditions, and salt stress to analyze the response of *A. argyi* to abiotic stress. Among the different stresses, the plants exhibited the strongest sensitivity to cold stress. The abiotic stress treatments also reduced eugenol synthesis. Several potential abiotic stress-tolerant genes that can be used to cultivate stress-tolerant *A. argyi* by polymerizing or generating transgenic plants were identified. Li X.Y. et al. (2020) used transcriptome sequencing to analyze the molecular mechanism underlying the responses of different tissues of *Salvia miltiorrhiza* to moderate drought stress. A total of 58,085 unigenes were identified, of which 28,846 were annotated as unigenes. Depending on the GO enrichment results, the differential transcripts of roots and leaves were significantly enriched in metabolic processes and catalytic activity. Under moderate drought stress, the expression of genes encoding key enzymes involved in the biosynthesis of phenylpropanoids and terpenoids was upregulated. The findings provided a scientific basis for further research on the mechanism underlying the biosynthesis of medicinal components of *S. miltiorrhiza* and methods for effective irrigation in cultivation. Wang L.R. et al. (2020) studied the transcriptome information of kernel apricot and found DEGs at normal and low temperatures. Using high-throughput sequencing, the transcriptome was sequenced at room temperature and low temperature, and 116,957 and 31,360 unigenes were identified. Twelve genes were expressed at higher levels at room temperature than at low temperatures, whereas 38 genes were expressed at higher levels at low temperatures than at room temperature. The results of this study provided valuable information for the breeding of cold-resistant varieties using genetic engineering. Feng et al. (2020) used the third-generation sequencing technology to sequence the full-length transcriptome of *Angelica sinensis* and used NGS technology to perform differential expression sequencing analysis of the transcriptome of wild-type and cultivated *A. sinensis*. A total of 25,463 differentially expressed transcripts were identified using NGS. Transcripts that differed were primarily associated with the plant–pathogen interaction pathway and plant hormone signal transduction. This study provided the basic information for the screening and cultivation of *A. sinensis*. Transcriptome sequencing technology was used to analyze the callus tissue of *S. laniceps* to identify the genes related to frost resistance (Xu, 2019). Through GO enrichment analysis, 155 terms related to survival at low temperatures were identified, including those associated with low-temperature response, oxidative stress response, and plant hormones. KEGG enrichment analysis revealed that pathways such as ribosome, fatty acid metabolism, and unsaturated fatty acid biosynthesis were significantly enriched during the low-temperature response. This study screened a large number of genes related to frost resistance in *S. laniceps*, and the findings provided a basis for subsequent research on the topic (Supplementary Data Sheet 6).

## Discussion

Transcriptome sequencing is currently one of the most popular sequencing technologies used in life science and can be used without genomic reference sequences. Transcriptomic analysis has a wide range of applications and has been used to study various medicinal plants. As a rapid, high-coverage, high-efficiency, and high-throughput analytical method used for obtaining genomic information on medicinal plants, the research applications of transcriptomics continue to expand, and some of the areas in which the method has been applied are mining of novel functional genes, investigation of the synthesis pathways of secondary metabolites, identification of plant developmental pathways (Huang et al., 2014), and obtaining useful information for the breeding and standardized cultivation of fine varieties of medicinal plants (Li H. et al., 2018). An improved understanding of the synthetic pathways of secondary metabolites of medicinal plants and related genes would strengthen the investigation of secondary metabolism regulatory networks and promote studies on secondary metabolism in medicinal plants.

To resist biological and abiotic stresses, some plants produce multiple secondary metabolites, some of which can be utilized to treat various human diseases. Owing to their medicinal value, these plants are usually referred to as medicinal plants. Currently, there are approximately 270,000 plant species recognized worldwide, of which fewer than 40,000 species exhibit putative medicinal value (Mamedov and Nazim, 2012; Tan et al., 2015). However, barring some model plants, which serve as useful research resources and sources of relevant genomic information, most plants are considerably unexplored with respect to genomic information. Owing to the lack of research on the characterization of medicinal plant transcriptomes, there exists a significant gap in plant genomic data, which hinders research on important topics, such as the identification of key DEGs and pathways associated with secondary metabolite synthesis, which require transcriptomic data. Therefore, research on medicinal plant transcriptomes should be prioritized to obtain data for subsequent research on plants (Wang and Yang, 2019). In the future, analysis and research based on transcriptome data will promote the discovery of new functional genes and secondary metabolic pathways.

The NGS technology allows us to have a deeper understanding of the complexity of the transcriptome. The shortcomings of NGS reads need to be assembled or reference genome, which limits its use in many transcriptomes research fields of medical plants. Due to the shortness read length of NGS technology, it is difficult to study the full-length transcript of medical plant, and usually only the local structure of the gene can be studied, and it is difficult to study alternative splicing events at the full-length transcript level. At present, with the development of sequencing technology, the application of third-generation sequencing technology in RNA-Seq research is increasing. However, due to the relatively expensive sequencing cost and low throughput of third-generation sequencing at this stage, it limits its use in transcription. At present, the NGS technology is mainly used, and the third generation is used as an auxiliary to carry out RNA-Seq research. There are also multi-omics technologies, such as genomics, metabolomics, and proteomics to carry out multi-omics joint research. At the same time, with the improvement of high sequencing throughput and the integration of multi-omics technologies, it is urgent to develop effective algorithms for data integration and mining, realize multi-omics integration, rapid and accurate analysis, and reveal that its biological functions are current research hotspots.

Traditional transcriptome sequencing technology analyzes the aggregated cells at the population level, and the results often reflect the dominant number of cells (Ranzoni et al., 2019). Due to the heterogeneity between cells, even if the phenotype is the same, the genetic information of the cells may be significantly different. Therefore, traditional transcriptome sequencing technology will cause a lot of low abundance information to be lost (Navin and Hicks, 2011). With the advancement of sequencing technology and the rapid decline of sequencing costs, the current single-cell transcriptome research is expected to enter a brand-new stage. Single-cell transcriptome sequencing can effectively supplement the heterogeneity of single-cell gene expression that is ignored by conventional sequencing, and it can systematically track the dynamic changes of single cells, thereby deepening the understanding of cell state, genetic essence and gene expression regulation of transcriptome, and promote the modernization of Chinese herbal medicine.

## Conclusion

The transcriptome sequencing technology primarily in use currently is an RNA-Seq method based on NGS, which has the advantages such as high throughput, high sensitivity, and high resolution. Although the third-generation sequencing has several advantages over NGS, such as long-read length, full transcript sequencing, and short time, the high mismatch rate of the third-generation sequencing has limited its application in transcriptome sequencing. However, the third-generation sequencing technology can be combined with NGS technology to correct errors and provide genotyping recognition. With the reducing costs of the third-generation sequencing and the increase in accuracy, the third-generation sequencing technology is expected to be used more frequently in transcriptome research to provide accurate and complete transcriptome sequencing results. Following the research model of economic crop, transcriptome sequencing is likely to be applied extensively in medicinal plant research. Currently, most research on medicinal plants is not limited to the use of RNA-Seq technology. Multi-omics that combine emerging metabolomics and proteomics technologies will be of prime importance in the development of transcriptomic technology. In the future, transcriptomics and multi-omics will promote the modernization of medicinal plant research.

## Author Contributions

JG and YL reviewed the literature and prepared the manuscript. JS and ZH reviewed the manuscript and created the images. XC supervised the work. All authors have read and approved the final manuscript.

## Conflict of Interest

ZH was employed by the company Yuxi Walvax Biotechnology Co., Ltd. The remaining authors declare that the research was conducted in the absence of any commercial or financial relationships that could be construed as a potential conflict of interest.

## Publisher’s Note

All claims expressed in this article are solely those of the authors and do not necessarily represent those of their affiliated organizations, or those of the publisher, the editors and the reviewers. Any product that may be evaluated in this article, or claim that may be made by its manufacturer, is not guaranteed or endorsed by the publisher.

## Supplementary Material

Supplementary Data Sheet 1 Comparison of the advantages and disadvantages of the next- and third-generation sequencing technologies.

Supplementary Data Sheet 2 Principle, advantages, and disadvantages of the traditional transcriptome sequencing method.

Supplementary Data Sheet 3 Summary of functional genes in the transcriptome of medicinal plants.

Supplementary Data Sheet 4 Summary of SSR markers in the transcriptome of medicinal plants.

Supplementary Data Sheet 5 Summary of secondary metabolic pathways in the transcriptome of medicinal plants.

Supplementary Data Sheet 6 Summary of research on medicinal plant transcriptome stress.
