# Supplementary material for: Research Progress and Future Development Trends in Medicinal Plant Transcriptomics
Source: Front Plant Sci. 2021 Jul 28;12:691838. doi: 10.3389/fpls.2021.691838 (PMC8355584; doi:10.3389/fpls.2021.691838)
Supplement: Supplementary Data Sheet 1 — Comparison of the advantages and disadvantages of the next- and third-generation sequencing technologies. [file Data_Sheet_1.docx]

**Supplementary Data sheet 1 Comparison of the advantages and disadvantages of next- and third-generation sequencing technologies**

| **Sequencing**  **technology** | **Principle** | **Advantage** | **Disadvantage** | **References** |
| --- | --- | --- | --- | --- |
| 454 pyrosequencing | Pyrosequencing technology | Long read length, large number of generated fragments and fast running. | May introduce deletions or insert sequencing errors and low throughput. | Zhang et al., 2016 |
| Solexa | Single molecule array technology | High throughput, low cost and suitable for detecting repetitive sequences. | Short read length makes De novo is difficult and Weak diversity. | Yao et al., 2017 |
| Solid | Sequencing by ligation technology | High accuracy, easy to distinguish between SNPs and sequencing errors. | Short read length, prone to chain decoding errors. | Xu, 2018 |
| SMRT | Sequencing by synthesis technology | Short time-consuming and no need for PCR amplification. | High cost and low throughput. | Li et al., 2018 |
| Nanopore | Electrical signal sequencing technology | High throughput, long read length, low cost and detect epigenetic regulation point. | High single-base error rate and long sequencing time. | Ma et al., 2019 |
