# Supplementary material for: Research Progress and Future Development Trends in Medicinal Plant Transcriptomics
Source: Front Plant Sci. 2021 Jul 28;12:691838. doi: 10.3389/fpls.2021.691838 (PMC8355584; doi:10.3389/fpls.2021.691838)
Supplement: Supplementary Data Sheet 2 — Principle, advantages, and disadvantages of the traditional transcriptome sequencing method. [file Data_Sheet_2.docx]

**Supplementary Data sheet 2 Principle, advantages, and disadvantages of the traditional transcriptome sequencing method**

| **Sequencing**  **technology** | **Principle** | **Advantage** | **Disadvantage** | **References** |
| --- | --- | --- | --- | --- |
| EST | Next-generation Sequencing technology | High gene separation efficiency, good versatility and high conservative, and low cost and easy operation. | Certain error in the data, incomplete genome information revealed, and requirements for library quality. | Zhang et al.,2012 |
| cDNA-AFLP | RT-PCR and AFLP technology | Good reproducibility, low false positives, can detect low-abundance mRNA, and accurately reflect the difference in expression between genes. | Many steps, time-consuming and costly, difficulty in operation and low recovery rat, and strict requirements and not easy to produce desired results. | Qian et al., 2012; Hang et al., 2006 |
| Chip technology | Hybridization technique | Fast, high throughput, diversity, automation and miniaturization. | High false positives and poor repeatability, the chip is expensive, experiment cost is high, it is only suitable for the detection of known sequences and no exploration of new genes. | Mironova et al., 2015; Wu et al., 2009; Su et al., 2016; Zhai and Lu, 2017 |
| SAGE | Sanger sequencing technology | Data is comparable, high resolution, low background noise, and easy to operate and scale. | Tags are susceptible to interference; sequencing is time-consuming and expensive. | Zhang and Sheng, 2008; Zhuo et al., 2015 |
| MPSS | Sanger sequencing technology | Good repeatability and high sensitivity, suitable for any organism, any traits, high experimental efficiency, and high genome coverage. | Requires more expensive hardware and matching software to work together. | Yang et al., 2009; Simkin et al., 2011 |
| RNA-seq | Next-generation and third-generation technology | Suitable for whole transcriptome analysis of any species, digital signal, high sensitivity, high accuracy and wide detection range. | Sequencing sequence read length is usually short, certain error rate, high false positives and the amount of data is too large to save. | Mironova et al., 2015; Cheng, 2004 |
