# Supplementary material for: Research Progress and Future Development Trends in Medicinal Plant Transcriptomics
Source: Front Plant Sci. 2021 Jul 28;12:691838. doi: 10.3389/fpls.2021.691838 (PMC8355584; doi:10.3389/fpls.2021.691838)
Supplement: Supplementary Data Sheet 3 — Summary of functional genes in the transcriptome of medicinal plants. [file Data_Sheet_3.docx]

**Supplementary Data sheet 3 Summary of functional genes in the transcriptome of medicinal plants**

| **Species** | **Sequencing platform** | **Number of transcripts** | **Other found** | **Breakthrough** |
| --- | --- | --- | --- | --- |
| *Vitis*  *amurensis* | Illumina GA-II | 6 850 DEGs (3676 cold-inducible and 3174 cold- repressed) | Find 855 AS events, 38 major *TF* families that were involved in cold regulation (326 genes). | Identified many new transcription factors (PLATZ, LIM, EIL and Nin-like, TUB, Whirly, and PcG) that were significantly affected by cold stress. |
| *Aloe*  *vera* | Illumina HiSeq | 161 733(root)  221 792(leaf) | unigenes:113 063(root)  141 310(leaf)  CDS:43,443(root)  43,178(leaf) | Transcription factors like AP2/ERF, bHLH and MYB and NAC were found to be involved in regulating secondary metabolism. |
| *Paeonia suffruticosa* | Illumina HiSeq 2000 | 81 725(unigenes) | 128(KEGG,  23 518 unigenes)  43 977(GO)  41 808(CDS)  14 768(COG, 25 classifications) | Find MYB, AP2/ERF, NAC, bHLH, RING- H, HSP and HIPP is related to drought stress responsive. |
| *Trillium govanianum* | Illumina GAIIx | 69 174 | 44 043(GO)  21 845(KOG)  13 525(DEGs)  3 553(KEGG, 5519 unigenes) | Identified array of CYP450s and UGTs can be good candidates for diversification of bioactive molecules. |
| *Polygonum minus* | Illumina HiSeq 2000 | 188 735 | 86 295(CDS)  1 109 309(GO terms, 53 functional groups)  474(KEGG, 28 643 unigenes) | 10 new enzymes involved in the flavonoid biosynthesis pathway were identified. |
| *Callerya speciosa* | Illumina RNA-seq | 161 926(unigenes) | 109 421(CDS)  87 126(GO, 56 functional groups)  47 162(COG, 25 functional categories)  69 375(KEGG)  4 538(DEGs)  54 transcription factors  gene families | Find many transcription factor gene families including bZIP,  GRAS, COL, MIKC, ERF, LBD, NAC and HB-other families may participate in sugar  and phytohormone signaling, flowering regulation, root development and cell expansion. |
| *Cornus officinalis* | Illumina HiSeq 4000 | 70 392 | 56 392(unigenes)  24 336(GO)  10 808(KOG, 25 categories)  18 435(KEGG, 371 path ways)  4 585(EDGs) | 5 864 unknown unigenes were obtained and fnd tow photosynthesis-related unigenes and four unigenes associated with chlorophyll a/b binding protein. |
| *Dracocephalum tanguticum* | Illumina RNA-seq | 187 447 | 151 463(unigenes)  86 496(KEGG, 362 path ways)  7 632 SSR | 22 predicted biosynthetic genes related to Rosmarinic acid from the transcriptome and two of these genes were identified as candidates. |
