# Supplementary material for: Research Progress and Future Development Trends in Medicinal Plant Transcriptomics
Source: Front Plant Sci. 2021 Jul 28;12:691838. doi: 10.3389/fpls.2021.691838 (PMC8355584; doi:10.3389/fpls.2021.691838)
Supplement: Supplementary Data Sheet 4 — Summary of SSR markers in the transcriptome of medicinal plants. [file Data_Sheet_4.docx]

**Supplementary Data sheet 4 Summary of SSR markers in the transcriptome of medicinal plants**

| **Species** | **SSR number** | **Summary** |
| --- | --- | --- |
| *Asparagus*  *officinalis* | 122 | SSR-15 amplified 8 alleles, find 2 genetic stocks contributing to the genetic makeup of all the 10 species. *Asparagus adscendens* and *Asparagus pyramidalis* were found closely related. |
| *Salvadora oleoides* | 21 055 | 101 SSRs were synthesized,94 of these has been successfully validated through PCR amplification and out of which 34 were found to be polymorphic. |
| *Panax vietnamensis* | 11 343 | Among the selected 101 primer pairs, 20 primer pairs were successfully amplified DNA fragments, and obvious  polymorphisms were observed. By analyzing the population structure and diversity of 9 polymorphic microsatellite loci, it is found that this population has a high level of genetic diversity. |
| *Gastrodia*  *elata* | 2 298 | Obtained 34,324 unigenes, of which 24,698 were annotated. 4236 unigenes were identified as TFs, and among 2298 SSRs, AG/CT repeat motif was the most frequent, a total of 498. |
| *Tinospora cordifolia* | 7 611 | Among all SSRs, 30 polymorphic SSRs were selected, and 230 alleles were amplified, which had high polymorphism. The results of AMOVA show that the genetic variation within the population is greater than that between the populations, which proves that there is a high genetic diversity. |
| *Menispermum* | 521 | 23 primer pairs were selected to evaluate the genetic diversity among *Menispermum* populations, showing high diversity. And the transferability was detected in the other three *Menispermaceae*, with a transferability rate of 85~90%. |
| *Paeonia lactiflora* | 21 998 | 45 pairs of primers with polymorphism were selected, and 290 alleles were obtained after amplification. Among them, SSR 43 has the most alleles amplified. The genetic diversity analysis of these 45 pairs of primers showed that they are highly polymorphic, which proves that *Paeonia lactiflora* has a high genetic diversity. |
