# Supplementary material for: Research Progress and Future Development Trends in Medicinal Plant Transcriptomics
Source: Front Plant Sci. 2021 Jul 28;12:691838. doi: 10.3389/fpls.2021.691838 (PMC8355584; doi:10.3389/fpls.2021.691838)
Supplement: Supplementary Data Sheet 5 — Summary of secondary metabolic pathways in the transcriptome of medicinal plants. [file Data_Sheet_5.docx]

**Supplementary Data sheet 5 Summary of secondary metabolic pathways in the transcriptome of medicinal plants**

| **Species** | **Active ingredients** | **Number of genes** | **Other found** | **Summary** |
| --- | --- | --- | --- | --- |
| *Entada phaseoloides* | Triterpene  saponins | 26 (cytochrome P450)  17 (uridine diphosphate glycosyltransferase) | 116 910(unigenes)  28 126(GO, 60 functional categories)  15 119(KEGG, 5 main categories)  11 812(COG, 31 sub-categories) | In the transcriptome, 8 β-amyrin synthase, 326 CYP450, and 148 UGT sequences were found. The high expression of β-amyrin synthase reveals the high concentration of triterpene sapogenin expression in the stem. 26 CYP450 and 17 UGT were up-regulated in the stem. |
| *Lantana*  *camara* | Phenylpropionic acid | 229 (leaf)  943 (root) | 20 044(DEGs)  Leaf:  72 877(unigenes)  36 209(GO, 25 groups)  18 853(KEGG, 5 main groups)  19 818(KOG, 26 protein groups)  Root:  513 985(unigenes)  255 964(GO, 57 sub-groups)  115 383(KEGG, 5 main groups)  155 979(KOG, 26 protein groups) | 49072 unigenes were found to be ubiquitously expressed in leave and root tissues. Among the 20044 DEGs, 11496 genes were up-regulated and 8548 genes were down-regulated. Through pathway analysis, it was found that 229 and 943 genes (coding for 17 and 19 enzymes) were involved in the synthesis of phenylpropanes pathway in leaf and root tissues, respectively. |
| *Tetrastigma hemsleyanum* | Anthocyanin | 14 (related genes)  16 (related metabolites) | 141 significant correlation combi-  nations between the genes and metabolites.  597 metabolites | TFs including MYB, WD40, and bHLH play an important role in the regulation of the anthocyanin pathway, and it is found that 8 WRKY are highly expressed in purple leaves. It is speculated that WRKY may be involved in the regulation of anthocyanin biosynthesis. |
| *Saussurea*  *lappa* | Sesquiterpenoid and flavonoid | 70 | 122 434(unigenes)  9 536(GO, 114 049 unigenes)  476(KEGG, 39 pathways)  11 639(SSRs, 10 323 unigenes) | 70 transcripts involved in the synthesis of flavonoid pathways, including phenylalanine ammonia-lyase (6 unigenes), cinnamate 4-hydroxylase (1 unigenes), 4-coumaroyl CoA-ligase (1 unigenes), chalcone synthase (3 unigenes), chalcone isomerase (2 unigenes), Flavanone 3-hydroxylase (7 unigenes), flavonol synthase (1 unigenes), flavanone 3′ hydroxylase (3 unigenes), dihydroflavonol reductase (1 unigenes), leucoanthocyanidin oxidase (3 unigenes) and UFGT (42 unigenes) . |
| *Arisaema heterophyllum Blume* | Isoflavones | 87 | 109 937(unigenes)  53 451(COG, 25 COG classes)  8 496(GO, 54 functional groups)  53 451(KEGG, 137 pathways)  31 094(DEGs)  28 537(SSRs, 21 200 unigenes) | It was found that tubers had the most up-regulated transcripts, and the number of unigenes involved in metabolic pathways in tubers was also the most. It was also found that 14,510 unigenes were uniquely expressed in tubers. Found the key enzymes CHS, CHI and IFS2 that code for biosynthesis of isoflavonoid. |
| *Ginkgo*  *biloba* | Flavonoids | 16 | 37 625(unigenes)  14 933(GO)  14 899(KOG, 25 groups)  11 267(KEGG, 22 pathways)  5 587(TFs, 83 families)  457(DEGs) | The 14 structural genes related to the flavonoid biosynthetic pathway were found to include a total of 53 non-specific genes. Many transcription factors related to the regulation of flavonoids have been found in *Abrus mollis*, and the MYB family plays an important regulatory role among them. |
| *Abrus*  *mollis* | Flavonoids | 52 | 53 743(unigenes)  35 656(KOG, 25 function classifications)  146 499(GO)  7 572(KEGG, 128 pathways)  5 214(SSRs, 4 428 unigenes) | Found multiple unigenes related to the biosynthetic pathway of flavonoids. 72 unigenes were identified to encode 23 enzymes involved in the biosynthesis pathways of phenylalanine, tyrosine, and tryptophan. And it was found that there are 204 unigenes in the phenylpropane biosynthetic pathway, 23 unigenes in the flavonoid biosynthetic pathway, and 22 unigenes in the isoflavone biosynthetic pathway. |
| *Artemisia*  *argyi* | Terpenoids | 251 | 67 446(unigenes)  20 091(GO, 54 functional categories)  44 750(KEGG, 135 pathways)  25 049(DEGs)  2 056(TFs, 59 families) | Discovered genes encoding encoding lectins28 and ribosome-inactivating proteins. The unigenes encoding PMK,  MVD, DXR, CMS, CMK and HDS were highly differentially expressed in leaves, and 461 candidate factors found may be related to the regulation of terpenoid biosynthesis |
| *Panax*  *ginseng* | Triterpene  saponins | cytochrome P450  UDP glycosyltransferase gene | Chunpoong:  35 527(transcripts)  26 423(GO)  Cheongsun:  27 716(transcripts)  21 096(GO) | In vitro culture conditions are a kind of stress to the adventitious roots of ginseng, which protect plants from damage by inducing stress-related genes, 10 transcripts encoding ginsenoside biosynthesis related enzymes were identified, and 21 transcripts related to UGT protein were also found. In addition, three transcripts that play important roles in the biosynthetic pathway of triterpene saponins have also been discovere. |
| *Panax notoginseng* | Ginsenoside and alkaloid | 270 | 107 340(unigenes)  9 908(KEGG, 135 pathways)  350(CYP450 families)  342(GT families) | A new gene was discovered, named CYP716A53v2, and sequence analysis proved that it was a full-length homolog of Panax notoginseng. It was found that the expression of this gene in roots was higher than that in leaves and flowers. 72 unigenes were identified and classified into 6 enzymes related to alkaloid biosynthesis. |
| *Panax notoginseng* | Notoginsenoside | 38 | 30 852(unigenes)  18 689(GO)  16 300(KEGG)  2 772(SSRs, 2361 unigenes)  906(TFs) | Some genes related to triterpene saponins biosynthesis were identified, including cytochrome P450s and glycosyltransferases. Through phylogenetic analysis, it was found that a few candidate CYP450 and UGT may be involved in triterpene saponins biosynthesis. Many transcription factors and SSR markers were also identified. |
