# Supplementary material for: Research Progress and Future Development Trends in Medicinal Plant Transcriptomics
Source: Front Plant Sci. 2021 Jul 28;12:691838. doi: 10.3389/fpls.2021.691838 (PMC8355584; doi:10.3389/fpls.2021.691838)
Supplement: Supplementary Data Sheet 6 — Summary of research on medicinal plant transcriptome stress. [file Data_Sheet_6.docx]

**Supplementary Data sheet 6 Summary of research on medicinal plant transcriptome stress**

| **Species** | **Environmental stress factors** | **Other found** | **Summary** |
| --- | --- | --- | --- |
| *Artemisia argyi* | Cold, drought, flood and salt stress | Unigenes:  64 603(control)  55 891(cold)  58 018(drought)  55 956(flood)  46 235(salt)  CDS:  40 560(control)  32 731(cold)  36 502(drought)  34 650(flood)  27 811(salt)  DEGs:  14 639(cold)  16 664(flood)  15 181(drought)  12 899(salt)  KEGG:  11 107(control)  9 678(clod)  10 201(flood)  8 073(drought)  6 890(salt)  31(TF families) | It is found that among the four stresses, low temperature stress has the greatest impact on plants, and under other stress conditions, plants can survive for 30 days. It was identified that the expression of CAD and CCOMT transcripts increased under stress conditions, which may be related to the lignin biosynthetic pathway. In addition, transcription factors MYB/MYC, WRKY and NAC play an important role in the expression of abiotic stress-related genes in this plant. |
| *Salvia miltiorrhiza* | Drought stress | 58 085(unigenes)  1 853(DEGs, 1 222 upregulate, 631 downregulate)  328(KEGG, terpenoid pathway)  1 128(TFs) | Studies have found that the response of Salvia miltiorrhiza to the early stage of drought stress may involve the entire plant. In the early stage of drought stress, the growth of the aerial parts of Salvia miltiorrhiza can be inhibited by inhibiting the sugar metabolism in the leaves of the salvia and inducing the programmed death of primary parietal cells. In addition, under moderate drought stress, transcription factors such as WRKY, AP2/ERF, GRAS, bHLH, bZIP, Dof and the up-regulated terpenoid genes in roots and leaves form a gene co-expression network, respectively. These transcription factors may be the key to moderate drought stress to promote the biosynthesis of salvia miltiorrhiza. |
| *Kernel Apricot* | Cold stress | 31 360(unigenes)  12 955(GO)  7 185(KEGG)  50 978(KOG) | Based on the found differentially expressed genes, cluster analysis of the top 50 genes with the largest variance in expression levels between treatments showed that 12 genes had higher expression levels at room temperature and lower expression levels in low-temperature treatments. The other 38 genes have higher expression levels in low temperature treatment and lower expression levels in room temperature. |
| *Angelica sinensis* | Drought, temperature and high salt stress | 113 906(unigenes)  60 638(GO)  42 876(KOG)  25 463(DEGs) | It is found that a large number of unigenes are involved in the metabolism of terpenoids and polyketides, amino acid compounds, starch and sucrose metabolism and other metabolic pathways. |
| *Saussurea laniceps* | Cold stress | 88 862(unigenes)  29 157(GO, 54 functional categories)  9 462(KEGG)  14 253(DEGs)  456(TFs) | AP2-EREBP, WRKF, BHLH, MYB and NAC five major transcription factor families play a key regulatory role in the response of plants to low temperature stress. From these five types of transcription factors, 32 transcription factors that are prominent at low temperature have been further locked. These transcription factors play an important role in responding to low temperature stress. |
